# Pediatric invasive device utility and harm: a multi-site point prevalence survey

**DOI:** 10.1038/s41390-023-03014-1

**Published:** 2024-01-11

**Authors:** Mari Takashima, Victoria Gibson, Eloise Borello, Lily Galluzzo, Fenella J. Gill, Sharon Kinney, Fiona Newall, Amanda J. Ullman

**Affiliations:** 1 School of Nursing, Midwifery and Social Work, The University of Queensland, St Lucia, QLD Australia; 2 Queensland Children’s Hospital, Children’s Health Queensland Hospital and Health Service, South Brisbane, QLD Australia; 3 Nursing Research Department, Royal Children’s Hospital, Melbourne, VIC Australia; 4 Perth Children’s Hospital, Child and Adolescent Health Service, Perth, WA Australia; 5 School of Nursing, Faculty of Health Sciences, Curtin University, Perth, WA Australia; 6 Department of Nursing and Paediatrics, The University of Melbourne, Melbourne, VIC Australia

## Abstract

**Background and aims:**

Invasive devices are widely used in healthcare settings; however, pediatric patients are especially vulnerable to invasive device-associated harm. This study aimed to explore invasive device utility, prevalence, harm, and clinical practice across three Australian pediatric tertiary hospitals.

**Methods:**

In 2022–2023, a multi-center, observational, rolling-point-prevalence survey was conducted. Fifty-per-cent of inpatients were systemically sampled by random allocation. Patients with devices were then followed for up to 3-days for device-related complications/failures and management/removal characteristics.

**Results:**

Of the 285 patients audited, 78.2% had an invasive device (*n* = 412 devices), with a median of 1 device-per-patient (interquartile range 1–2), with a maximum of 13 devices-per-patient. Over half of devices were vascular access devices (*n* = 223; 54.1%), followed by gastrointestinal devices (*n* = 112; 27.2%). The point-prevalence of all device complications on Day 0 was 10.7% (44/412 devices) and period-prevalence throughout the audit period was 27.7% (114/412 devices). The period-prevalence of device failure was 13.4% (55/412 devices).

**Conclusions:**

The study highlighted a high prevalence of invasive devices among hospitalized patients. One-in-ten devices failed during the audit period. These findings underscore the need for vigilant monitoring and improved strategies to minimize complications and enhance the safety of invasive devices in pediatric hospital settings.

**Impact:**

- A high prevalence of invasive devices among hospitalized patients was reported. Of the 285 patients audited, almost 80% had an invasive device (total 412 devices), with a median of 1 device-per-patient and a maximum of 13 devices-per-patient.
- The most common devices used in pediatric healthcare are vascular access devices (*n* = 223; 54.1%), however, 16% (*n* = 36) of these devices failed, and one-third had complications.
- The point prevalence of all device complications at day 0 was 10.7% (44 out of 412 devices), with a period prevalence of 27.7% (114 out of 412 devices) throughout the audit period.

## Introduction

Invasive devices are essential for the treatment and management of patients across all healthcare settings.^1^ They are used as a pathway to deliver or remove fluids and gases, including medications, nutrition, body fluid and respiratory gases.^1–3^ Common invasive devices include intravascular catheters, urinary catheters, endotracheal tubes and nasogastric tubes.^1,2^ Invasive devices provide a portal of entry to the body, meaning their use carries an inherent risk of patient harm, including infections. Globally, healthcare-associated infections (HAI) are the most common adverse event occurring in healthcare settings.^4^ These infections can be systemic (i.e., bloodstream infections), affect a single organ (e.g., pneumonia), or occur at the insertion site (e.g., tissue infection). The use of invasive devices magnifies both the risk and impact of healthcare-associated infections.^1,3^ Other forms of harm associated with the use of invasive devices include mechanical damage to the area of insertion and dwell, resulting in venous thromboembolism (e.g., deep vein thrombosis), mechanical trauma (e.g., bleeding at the insertion site), and pressure injuries. Additionally, the device can dysfunction during treatment, becoming blocked or dislodged, causing planned treatment to be interrupted or prematurely ceased. These non-infectious invasive device-associated harms are also associated with increased morbidity, mortality and considerable healthcare costs.^5,6^

While the incidence of HAIs are thought to be under-reported,^1^ pediatric patients (≤18 years) are at greater risk than adults of developing certain types of HAI such as central line-associated bloodstream infections (CLABSIs).^3^ The prevalence of other types of device-associated harm in pediatrics, such as pressure injuries, is even more unclear, with predominantly only small, single-center studies published.^7^ Reducing harm caused by invasive devices is a key priority of the Australian Commission on Safety and Quality in Health Care’s National Safety and Quality Health Service Standards. This can be achieved by ensuring the appropriate selection, insertion, management and timely removal of invasive devices.^8,9^ However, practices commonly vary between institutions, often due to a lack of research to guide healthcare decision-making. The World Health Organization and other patient safety organizations strongly recommend national surveillance of infections, pressure injuries and other forms of healthcare-associated harm with timely data feedback and benchmarking capacity.^9,10^ Describing the current state of Australian pediatric healthcare is a vital step towards the development of high-quality research into reducing the harm associated with invasive device use in pediatrics. This will allow the demonstration of the relative burden of the utility of these devices and assist in the prioritization of future research programs.

The primary objectives of the study were to identify the prevalence of invasive device utility in Australian pediatric healthcare facilities, to estimate the prevalence of complications associated with these invasive devices, and describe the management practice including documentation, dressing integrity, and pain.

## Methods

### Study design

**Fig. 1:**
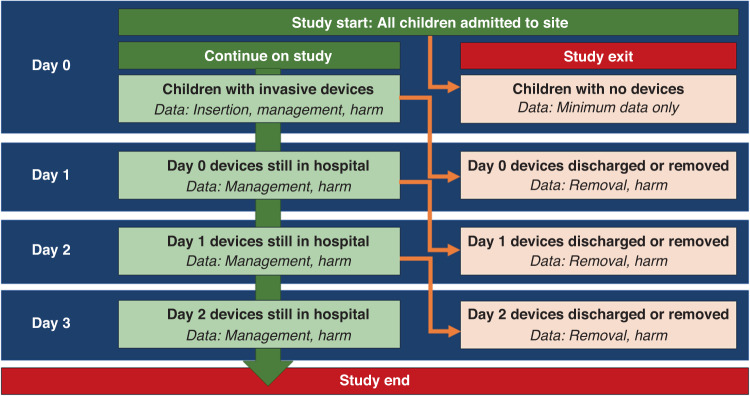
Study schema.

A multicenter, observational, rolling point prevalence survey was conducted across three Australian pediatric tertiary hospitals (Queensland Children’s Hospital, Perth Children’s Hospital and the Royal Children’s Hospital in Melbourne). All of these hospitals are standalone, tertiary pediatric hospitals caring for children in three large states (Queensland, Western Australia, and Victoria) with a total of 1007 beds. Hospitals were recruited via expression of interest and convenience sampling. The methods are based on a modified version of the European Centre for Disease Prevention and Control (ECDC) methodology for point prevalence surveys on healthcare-associated infections,^11^ recently operationalized in Australia.^4^ The initial audit day (Day 0) was chosen by the local site investigators within a fixed schedule (November 2022 to February 2023), based on the availability of staff. After the initial audit (Day 0), follow-up audits occurred daily for three days (Days 1, 2, 3) to assess for device removal, complications and deviations in recommended care. Only previously audited patients/devices were assessed in the follow-up phase (see Fig. 1).

### Setting and population

#### Ward and patient selection

All acute care inpatient wards were included, and all outpatient (e.g., hospital in the home), non-admitted emergency departments, and mental health units were excluded. Any child or adolescent (0-18 years) admitted to the study wards before or at 8am on the first survey day (Day 0), and not discharged from the ward at the time of survey were eligible. Patients who were in the operating theater or on a day pass, experiencing an emergency code, receiving end-of-life care, or for any other reason for which auditing such patients was deemed unreasonable were excluded. For feasibility, and based on similar prevalence audits, from this cohort, we systematically sampled 50%, based on random allocation of odd or even bed numbers.^4^ Demographic data was collected for all patients in the allocated beds, and entered into a screening log to enable an accurate estimate of prevalence as a proportion of the total hospitalized population (i.e., denominator).

All patients with an invasive device progressed to the main part of the study. Invasive devices included any medical device that is inserted into the patient’s body, with a part of the device remaining outside of the body.^12^ Common devices include:

- Intravascular access devices: including venous and arterial, peripheral and central (including extracorporeal membrane oxygenation cannula, hemodialysis).
- Gastric devices: including gastric tubes (naso-, oro-, jeju-), and percutaneous endoscopic tubes.
- Respiratory support devices: including endotracheal tubes, tracheostomy, nasopharyngeal.
- Drains: including external ventricular drain, intercostal catheters, general wound drains, peritoneal dialysis.
- Urinary catheters: including intermittent, indwelling, and suprapubic catheters.
- Epidural catheters.
- Subcutaneous devices
- Pacing wires.

As described in Fig. 1, patients without an invasive device or a device not fitting this criterion (e.g., totally implanted device that is not accessed) were discontinued from the audit and had their initials, age, location/department, and primary diagnosis on a screening log to enable an accurate estimate of prevalence as a proportion of the total hospitalized population (i.e., denominator). In this study, we adopted a purely descriptive design, focusing on observing, recording, and describing the findings without any prior sample size calculation. While our study was primarily descriptive in nature, we made a concerted effort to enhance the generalizability of our findings by systematically sampling 50% of the study population by random allocation and following the previous rigorous and well-established framework by ECDC.^11^

### Outcomes

#### Primary outcomes

1. **Utility:** Number and type of invasive devices in situ per patient and per 100 patients.
2. **Harm:** Presence of invasive device-associated complications in the prior 24 h (as a group, and individually; assignment by infectious disease expert); defined as:

#### HAI

- Blood stream infections: including central line-associated blood stream infections (established based on European Centre of Disease Prevention and Control criteria,^11^ i.e., including laboratory confirmation).
- Tissue infections: including surgical site infection (established based on European Centre of Disease Prevention and Control criteria,^11^ i.e., including laboratory confirmation).
- Organ infections: including pneumonia, urinary tract, gastro-intestinal infections (established based on European Centre of Disease Prevention and Control criteria,^11^ i.e., including laboratory confirmation).

#### Mechanical injuries

- Venous thromboembolism: including pulmonary embolism, deep vein thrombosis (symptomatic [e.g., limb swelling],^13^ radiologically diagnosed vessel thrombosis adjacent to the device, as assigned by radiologist^14^).
- Pressure injuries: Graded as per National Pressure Ulcer Advisory Panel, European Pressure Ulcer Advisory Panel and Prevention and Treatment of Pressure Ulcers/Injuries guidelines.^15^
- Other device-associated skin complications: including wound dehiscence, contact and allergic dermatitis, and skin tear surrounding device insertion area.^16^
- Bleeding/hematoma: at insertion site.
- Other mechanical trauma: including infiltration and extravasation injuries.
- Pain: presence of device-related pain by patients, parents, and clinicians.

#### Device dysfunction

- Complete device occlusion/blockage: including infiltration and extravasation.
- Device dislodgement: complete or partial dislodgment.

#### Secondary outcomes

1. *Insertion characteristics*: Invasive device characteristics (gauge, number of lumens, current usage), reason for invasive device insertion, evidence of documentation
2. *Management characteristics*: Dressing integrity, evidence of documentation
3. *Removal characteristics*: Reason for device removal (completion of treatment, transfer, device dysfunction)

### Data collection

A local primary coordinating investigator at each participating healthcare facility was chosen based on their clinical and research experience and facilitated the audit organization and implementation. All audit staff were clinicians within the healthcare facility and were provided training in data collection methodology and use of data collection tools by the local coordinating investigators prior to the audit. Core training materials were standardized with further tailoring to be appropriate to each site. Each audit day, data quality checks were performed with all audit staff using case scenarios. Data was collected using mobile devices and entered into a secure online web-based survey tool (REDCap: Research Electronic Data CAPture; Vanderbilt, United States of America; http://project-redcap.org/). Senior clinicians were available at each site on the audit days for additional support as required, and assignments of infection and thrombosis were checked by relevant experts using pathology results and radiology reports.

Patient-level data, including demographic and clinical characteristics, were prospectively collected for descriptive purposes (i.e., age, primary diagnosis, sex, Aboriginal/Torres Strait Islander status, language, and country of birth) at the time of the visit to the ward. The auditors had access to patient medical records, pathology, and microbiology databases. Auditors were advised to seek clarification from clinicians at the bedside if the information held in the medical records was not clear.

The audit team prospectively collected data through observation and discussion with patients and their families. All invasive devices were visually observed by the audit team to assess the primary and secondary outcomes. Further information was collected from the medical record, including insertion documentation and the frequency of nursing and medical documentation of the device in the previous 24 hours. Where specific information was missing or not available, this was captured in the audit to understand documentation practices and compliance.

As demonstrated in the prior study scheme (Fig. 1), follow-up audits were undertaken every 24 hours for up to 3 days. If the patient was discharged, follow-up ceased. If the device had recently been removed, the site was visualized to ascertain harm outcomes. For those with the device still in situ, the site was assessed, as above.

### Data analysis

The demographic and device characteristics of the participants are descriptively reported, using categorical and continuous descriptors appropriate to their distribution. The device utility per 100 persons was calculated by dividing the number of patients who utilized the device divided by the total number of patients and then multiplying it by 100. If a patient had multiple instances of the same device, it was counted as a single patient in the calculation. The prevalence of complications was calculated by dividing the number of patients with complications by the total number of assessed patients by device type. Data were analyzed using the Stata Statistical Software: Release 15 (College Station, TX: StataCorp LP).

### Interrater reliability

Inter-rater reliability for the data collection process^17^ was tested using a small group of auditors and patients at each location prior to the broader audit. Each site had four auditors conduct the inter-rater reliability assessment. Two sites utilized audit nurses with different skill and experience levels (junior and senior nurses) and one site used only junior-level nurses. Four auditors consecutively assessed four patients and completed the demographic and device page for Day 0. Gwet’s AC was calculated instead of kappa score due to kappa paradox from extremely high agreements. Overall percent agreement for the demographic section was 0.77 (0.49–1.00), and Gwet’s AC was 0.74 (0.39–1.00), which had a substantial extent of agreement (Supplementary Table 1). Overall percent agreement for the device section was 0.88 (0.70–1.00), and Gwet’s AC was 0.87 (0.65–1.00), which had an almost perfect agreement. The data from the inter-rater reliability assessments were not included in the final results.

### Ethical consideration

The study was approved by the Children’s Health Queensland Hospital and Health Service Human Research Ethics Committee (HREC/22/QCHQ/83875) and the University of Queensland (2022/HE000443). Site-specific authorization was granted for each participating hospital.

## Results

### Participant characteristics

**Fig. 2:**
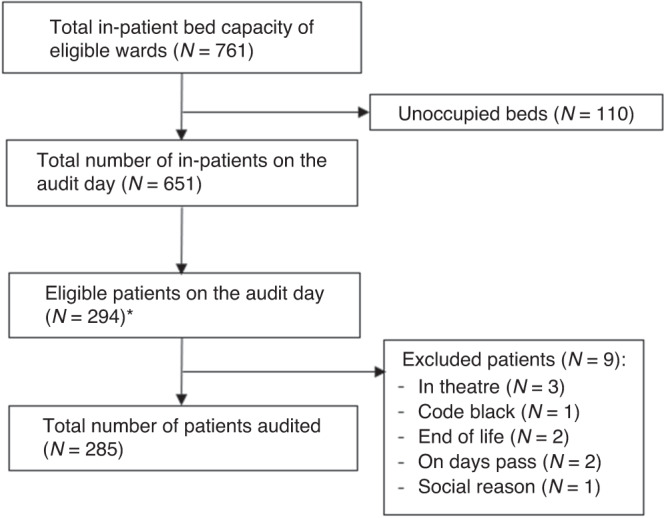
Process of patient selection (On arrival to the ward, the research assistants visited every odd or even numbered bed (according to the random allocation). *50% of patients were sampled, based on random allocation of odd or even bed numbers.

A total of 285 patients were included in the survey (Fig. 2). Demographic variables are described in Table 1. The median age of patients was 5 years of age, ranging from 0 to 17 (interquartile range [IQR]: 0.83–11). Of the sample patients, 147 (51.6%) were female, and 138 (48.4%) were male. The majority of patients identified as neither Aboriginal or Torres Strait Islander (*n* = 256; 89.8%), and 7.7% (*n* = 22) were Aboriginal and/or Torres Strait Islander. English was the most commonly used first language (*n* = 271; 95.1%), followed by Asian languages (*n* = 14; 4.9%), African/Middle Eastern Languages (*n* = 9; 3.2%), and Aboriginal and/or Torres Strait Islander languages and European (excluding English) languages (*n* = 4; 1.4%) each. Around 10% of patients (*n* = 25) were born outside Australia. The admission sources were mostly emergency/unplanned (*n* = 199; 69.8%), followed by medical-booked admission (n = 50; 17.5%) and surgical-booked admission (*n* = 36; *n* = 12.6%). About one-third of the participants were admitted for general medical diagnosis (*n* = 99; 34.7%), followed by respiratory diagnosis (*n* = 49; 17.2%) and oncology/hematology (*n* = 43; 15.1%). The median length of stay from admission to audit day was 5 (IQR: 2–14) days.

**Table 1:** Participant characteristics (*N* = 285 patients).

Variables		Medical *N* (%)	Surgical *N* (%)	Critical Care *N* (%)	Mixed medical and surgical *N* (%)	Total *N* (%)
		*N* = 135	*N* = 48	*N* = 58	*N* = 109	*N* = 285
Age (Years)	Median (IQR)	6 (1.8–12)	5 (1.6–10)	0.3 (0.1–4)^a^	8 (1.2–13)	5 (0.83–11)
Sex	Female	67 (49.6)	29 (60.4)	27 (46.6)	24 (54.6)	147 (51.6)
	Male	68 (49.6)	19 (39.6)	31 (53.5)	20 (45.5)	138 (48.4)
Aboriginal and Torres Strait Islander Status^b^	Neither	123 (91.1)	43 (89.6)	47 (81.0)	43 (97.7)	256 (89.8)
	Australian Aboriginal	10 (7.4)	5 (10.4)	5 (8.6)	1 (2.3)	21 (7.4)
	Unknown/not stated	2 (1.5)	0 (0.0)	5 (8.6)	0 (0.0)	7 (2.5)
	Torres Strait Islanders	0 (0.0)	0 (0.0)	1 (1.7)	0 (0.0)	1 (0.4)
Language^b^	English	130 (96.3)	46 (95.8)	55 (94.8)	40 (90.9)	271 (95.1)
	Asian languages	4 (2.9)	2 (4.2)	4 (6.9)	4 (9.1)	14 (4.9)
	African/Middle Eastern languages	5 (3.7)	0 (0.0)	2 (3.5)	2 (4.6)	9 (3.2)
	Aboriginal and/or Torres Strait Islander languages	0 (0.0)	2 (4.2)	1 (1.7)	1 (2.3)	4 (1.4)
	European (excluding English) languages	3 (2.2)	0 (0.0)	1 (1.7)	0 (0.0)	4 (1.4)
	Pacific Islander languages	1 (0.7)	1 (2.1)	0 (0.0)	0 (0.0)	2 (0.7)
	Arabic languages	0 (0.0)	0 (0.0)	1 (1.7)	0 (0.0)	1 (0.4)
Country of Birth	Australia	115 (85.2)	47 (97.9)	57 (98.3)	41 (93.2)	260 (91.2)
	Other/Unknown	11 (8.1)	0 (0.0)	0 (0.0)	0 (0.0)	11 (3.9)
	Asian countries	5 (3.7)	0 (0.0)	1 (1.7)	2 (4.6)	8 (2.8)
	United Kingdom	2 (1.5)	0 (0.0)	0 (0.0)	0 (0.0)	2 (0.7)
	New Zealand	2 (1.5)	0 (0.0)	0 (0.0)	0 (0.0)	2 (0.7)
	Africa and Middle Eastern countries	0 (0.0)	0 (0.0)	0 (0.0)	1 (2.3)	1 (0.4)
	Pacific Islands	0 (0.0)	1 (2.1)	0 (0.0)	0 (0.0)	1 (0.4)
Admission Source	Emergency/Unplanned	103 (76.3)	33 (68.8)	37 (63.8)	26 (59.1)	199 (69.8)
	Medical – booked admission	31 (22.9)	1 (2.1)	8 (13.8)	10 (22.7)	50 (17.5)
	Surgical – booked admission	1 (0.7)	14 (29.2)	13 (22.4)	8 (18.2)	36 (12.6)
Admission related diagnosis^b^	General Medical	61 (45.2)	3 (6.3)	23 (39.7)	12 (27.3)	99 (34.7)
	Respiratory	33 (24.4)	0 (0.0)	12 (20.7)	4 (9.1)	49 (17.2)
	Oncology/Hematology	38 (28.2)	3 (6.3)	2 (3.5)	0 (0.0)	43 (15.1)
	General surgical	4 (2.9)	29 (60.4)	6 (10.3)	3 (6.8)	42 (14.7)
	Cardiac	4 (2.9)	0 (0.0)	23 (39.7)	12 (27.3)	39 (13.7)
	Gastroenterology	12 (8.9)	7 (14.6)	5 (8.6)	7 (15.9)	31 (10.9)
	Neurology	10 (7.4)	3 (6.3)	4 (6.9)	7 (15.9)	24 (8.4)
	Orthopedics	3 (2.2)	10 (20.8)	0 (0.0)	1 (2.3)	14 (4.9)
	Trauma	7 (5.2)	2 (4.2)	1 (1.7)	1 (2.3)	11 (3.9)
	Sepsis	4 (2.9)	0 (0.0)	6 (10.3)	0 (0.0)	10 (3.5)
	Mental Health	4 (2.9)	0(0.0)	0 (0.0)	4 (9.1)	8 (2.8)

*IQR* interquartile range, *N* number, *NICU* neonatal intensive care unit.

^a^Includes 16 neonates from NICU.

^b^Participants can have multiple options.

### Device utility and characteristics

A total of 412 devices were audited (Supplementary Table 2). The median number of devices per patient was 1 (IQR 1–2), and ranged between 0 and 13 devices. More devices were used per patient in critical care settings (median 2; IQR 1–3), then followed by surgical and medical (median 1; IQR 0–2), and mixed settings (median 1; IQR 0–1). Approximately 21.8% of patients (*n* = 62) had no devices.

The flow of devices during the audit period is reported in Supplementary Fig. 1. About half of the devices were removed, or patients were discharged with a device by the end of the audit period. About half of the devices were vascular access devices (*n* = 223; 54.1%), with peripheral intravenous catheters (PIVC) being the most prevalent (*n* = 114; 27.7%). Within central venous access devices (*n* = 94; 22.8%), there were similar numbers of peripherally inserted central catheters (PICC; *n* = 29; 7.0%) and tunneled cuffed catheters (*n* = 30; 7.3%). Gastrointestinal devices were the second most prevalent (*n* = 112; 27.2%), with the majority being nasogastric tubes (*n* = 89; 21.6%). Following this are drains (*n* = 25; 6.1%) and urinary devices (*n* = 20; 4.9%). The number of devices audited was 412 devices on Day 0, 364 devices on Day 1, 287 devices on Day 2, and 225 devices on Day 3.

Device utility rate (Table 2) of vascular access devices was highest in surgical ward settings (79.2 per 100 patients), followed by critical care settings (77.6 per 100 patients). The utility rate of gastrointestinal devices, respiratory devices, drains, and urinary devices was highest in critical care settings (62.1, 22.4, 15.5, 15.5 per 100 patients, respectively).

**Table 2:** Utility of invasive devices (*N* = 412 devices).

	Medical		Surgical		Mixed medical and surgical^a^		Critical Care		Total	
Device type	*N* (%) *N* = 145	Device utility per 100 patients^b^	*N* (%) *N* = 69	Device utility per 100 patients^b^	*N* (%) *N* = 40	Device utility per 100 patients^b^	*N* (%) *N* = 158	Device utility per 100 patients^b^	*N* (%) *N* = 412	Device utility per 100 patients^b^
Vascular access devices	88 (60.7)	60.7	46 (66.7)	79.2	20 (50.0)	16.5	69 (43.7)	77.6	223 (54.1)	64.2
Gastrointestinal devices	44 (30.3)	32.6	7 (10.1)	12.5	18 (45.0)	16.5	43 (27.2)	62.1	112 (27.2)	36.5
Respiratory/airway	2 (1.4)	1.5	1 (1.5)	2.1	0 (0.0)	0.0	13 (8.2)	22.4	16 (3.9)	5.6
Drains	2 (1.4)	1.5	6 (8.7)	10.4	1 (2.5)	0.9	16 (10.1)	15.5	25 (6.1)	6.0
Urinary	3 (2.1)	2.2	7 (10.1)	14.6	1 (2.5)	0.9	9 (5.7)	15.5	20 (4.9)	7.0
Epidural infusion	0 (0.0)	0.0	1 (1.5)	2.1	0 (0.0)	0.0	0 (0.0)	0.0	1 (0.2)	0.4
Regional/local infusion	0 (0.0)	0.0	1 (1.5)	2.1	0 (0.0)	0.0	0 (0.0)	0.0	1 (0.2)	0.4
Cardiac pacing wires	0 (0.0)	0.0	0 (0.0)	0.0	0 (0.0)	0.0	2 (1.3)	1.7	2 (0.5)	0.4
Subcutaneous infusion	6 (4.1)	4.4	0 (0.0)	0.0	0 (0.0)	0.0	2 (1.3)	1.7	8 (1.9)	2.5
Extracorporeal membrane oxygenation	0 (0.0)	0.0	0 (0.0)	0.0	0 (0.0)	0.0	1 (0.6)	1.7	1 (0.2)	0.4
Cardiac lines	0 (0.0)	0.0	0 (0.0)	0.0	0 (0.0)	0.0	2 (100.0)	3.4	2 (0.5)	0.7
Ventricular assist device	0 (0.0)	0.0	0 (0.0)	0.0	0 (0.0)	0.0	1 (0.6)	1.7	1 (0.2)	0.4

^a^Defined as per site investigators.

^b^Device Utility per 100 patients = (Number of patients who utilized the device/Total number of patients) *100.

The device characteristics are reported in Supplementary Tables 3–10. The majority of the peripheral vascular access devices were PIVCs (*n* = 114; 88.3%), followed by arterial catheters (*n* = 8; 6.2%) abd midline catheters (*n* = 7; 5.4% Supplementary Table 3). These catheters are mostly placed on the hand, wrist, and forearms, but a substantial number of catheters were inserted in the antecubital fossa (PIVC: 21; 18.4%; midline 1; 14.3%; arterial line: 1; 12.5%). There were 94 central venous access devices (Supplementary Table 4), of which the majority were tunneled cuffed catheters (*n* = 30; 31.9%), followed by PICCs (*n* = 29; 30.8%), non-tunneled catheters (*n* = 14; 14.9%) and totally implanted device (*n* = 13; 13.8%). Most PICCs (*n* = 28; 96.6%), ports (n = 13; 100.0%), tunneled cuffed (*n* = 30; 100.0%), tunneled non-cuffed (*n* = 4; 100.0%) had 1–2 lumens. The majority of non-tunneled catheters had 3 lumens (*n* = 13; 92.9%).

For gastrointestinal devices (Supplementary Table 5), the most common device was the nasogastric/transpyloric device (*n* = 89; 79.5%), followed by the low-profile button (e.g., Mic-key) (*n* = 9; 8.0%), and percutaneous endoscopic gastrostomy device (*n* = 7; 6.3%). For the respiratory devices (*n* = 16; Supplementary Table 6), the most common device was the endotracheal device (*n* = 10; 62.5%), followed by tracheostomy tube (*n* = 4; 25.0%) and nasopharyngeal stents (*n* = 2; 12.5%). The characteristics of drain devices, urinary devices, epidural, regional, subcutaneous lines, cardiac pacing wires, cardiac lines, ventricular assist devices and extracorporeal membrane oxygenation are summarized in Supplementary Tables 7–10.

### Device complications

The point prevalence of complications across all devices on day 0 was 10.7% (44 out of 412 devices; Table 3). Highest complication point prevalence was experienced in drains (*n* = 5, 20%) followed by vascular access devices (*n* = 30, 13.5%). The period prevalence of all device complications throughout the audit period was 27.7% (114 out of 412 devices) and an incidence rate of 12.12 (95% confidence interval (CI): 10.28–14.29) per 100 device days. The period prevalence of device failure throughout the audit period was 13.4% (55 out of 412 devices), and the incidence rate was 4.73 (95% CI: 3.63–6.16) per 100 device days. Besides the ventricular assist device, where only one was audited and had a complication, the highest period prevalence of complications were urinary devices (*n* = 7, 35.0%), vascular access devices (*n *= 74, 33.2%), followed by drains (*n* = 7, 28.0%). Most common occurring complications for both point and period prevalence were bleeding and oozing from the insertion site in vascular access devices (*n *= 8; 3.6% and *n* = 18; 8.1%, respectively).

**Table 3:** Device-related harm by device types across the audit period (*N* = 412 devices).

		Prevalence *N* (%)		Incidence rate during the audit period	
Device type	Complication type	Point Prevalence (day 0)	Period prevalence (any day)	IR per 100 days (95% CI)	
All device	*N*	412	412	Audit days	1163
	Device Failure	NA	55 (13.4)	Device Failure	4.73 (3.63–6.16)
	Any complication	44 (10.7)	114 (27.7)	Any complications	12.12 (10.28–14.29)
Vascular access devices	*N*	223	223	Audit days	584
	Device failure	NA	36 (16.1)	Failure	6.16 (4.45–8.54)
	Any complication	30 (13.5)	74 (33.2)	Any Complications	12.67 (10.09–15.91)
	Bleeding and oozing from insertion site	8 (3.6)	18 (8.1)	Bleeding and oozing from insertion site	3.08 (1.94–4.89)
	Bruising	4 (1.8)	5 (2.2)	Bruising	0.86 (0.36–2.06)
	Catheter fracture	1 (0.4)	2 (0.9)	Catheter fracture	0.34 (0.09–1.37)
	Device and dressing-related skin injury	4 (1.8)	8 (3.6)	Device and dressing-related skin injury	1.37 (0.69–2.74)
	Dislodgement	2 (0.9)	11 (4.9)	Dislodgement	1.88 (1.04–3.40)
	Infiltration/Extravasation	2 (0.9)	10 (4.5)	Infiltration/Extravasation	1.71 (0.92–3.18)
	Leaking	4 (1.8)	14 (6.3)	Leaking	2.40 (1.42–4.05)
	Occlusion	1 (0.4)	11 (4.9)	Occlusion	1.88 (1.04–3.40)
	Edema around the site	0 (0.0)	1 (0.4)	Edema around the site	0.17 (0.02–1.22)
	Phlebitis	0 (0.0)	3 (1.3)	Phlebitis	0.51 (0.17–1.59)
	Pressure injury	2 (0.9)	3 (1.3)	Pressure injury	0.51 (0.17–1.59)
	Suspected BSI [confirmed]	0 [0] (0.0)	3 (1.3) [0]	Suspected BSI [confirmed]	0.51 (0.17–1.59)
	Suspected catheter thrombosis [confirmed]	1 [1] (0.4)	1 (0.4) [1]	Suspected catheter thrombosis [confirmed]	0.17 (0.02–1.22)
	Suspected local infection [confirmed]	1 [0] (0.4)	6 (2.7) [0]	Suspected local infection [confirmed]	1.03 (0.46–2.29)
	Unable to assess^a^	5 (2.2)	8 (3.6)	Unable to assess^a^	1.37 (0.69–2.74)
Gastrointestinal devices	*N*	112	112	Audit days	358
	Device failure	NA	11 (9.8)	Device Failure	3.07 (1.70–5.55)
	Any complication	7 (6.3)	21 (18.8)	Any Complications	5.87 (3.82–8.99)
	Catheter fracture	0 (0.0)	3 (2.7)	Catheter fracture	0.84 (0.27–2.60)
	Device and dressing related skin injury	4 (3.6)	11 (9.8)	Device and dressing-related skin injury	3.07 (1.70–5.55)
	Dislodgement	0 (0.0)	9 (8.0)	Dislodgement	2.51 (1.31–4.83)
	Occlusion	1 (0.9)	1 (0.9)	Occlusion	0.28 (0.04–1.98)
	Pressure injury	2 (1.8)	2 (1.8)	Pressure injury	0.56 (0.14–2.23)
	Unable to assess^a^	2 (1.8)	2 (1.8)	Unable to assess^a^	0.56 (0.14–2.23)
Respiratory/airway	*N*	16	16	Audit days	50
	Device failure	NA	1 (6.3)	Device Failure	2.0 (0.28–14.19)
	Any complication	1 (6.3)	2 (12.5)	Any Complications	4.0 (1.00–15.99)
	Cuff spontaneously deflates	0 (0.0)	1 (6.3)	Cuff spontaneously deflates	2.0 (0.28–14.19)
	Suspected VAP [confirmed]	1 [1] (6.3)	1 (6.3) [1]	Suspected VAP [confirmed]	2.0 (0.28–14.19)
Drains	*N*	25	25	Audit days	75
	Device failure	NA	0 (0.0)	Device Failure	0 (0.00–0.00)
	Any complication	5 (20.0)	7 (28.0)	Any Complications	9.33 (4.45–19.58)
	Leakage	3 (12.0)	4 (16.0)	Leakage	5.30 (2.01–14.21)
	Occlusion/poor drainage	2 (0.5)	2 (8.0)	Occlusion/poor drainage	2.66 (0.67–10.66)
	Suspected local infection [confirmed]	0 [0] (0.0)	1 (4.0) [1]	Suspected local infection [confirmed]	1.33 (0.19–9.47)
Urinary	*N*	20	20	Audit days	48
	Device failure	NA	5 (25.0)	Device Failure	10.42 (4.34–25.02)
	Any complication	1 (5.0)	7 (35.0)	Any Complications	14.58 (6.95–30.59)
	Damaged catheter	0 (0.0)	1 (5.0)	Damaged catheter	2.08 (0.29–14.79)
	Device and dressing-related skin injury	0 (0.0)	1 (5.0)	Device and dressing-related skin injury	2.08 (0.29–14.79)
	Dislodgement	0 (0.0)	4 (20.0)	Dislodgement	8.33 (3.13–22.20)
	Occlusion	1 (5.0)	1 (5.0)	Occlusion	2.08 (0.29–14.79)
Epidural infusion	*N*	1	1	Audit days	4
	Device failure	NA	0 (0.0)	Device Failure	0 (0.00–0.00)
	Any complication	0 (0.0)	0 (0.0)	Any Complications	0 (0.00–0.00)
Regional/local infusion	*N*	1	1	Audit days	3
	Device failure	NA	0 (0.0)	Device Failure	0 (0.00–0.00)
	Any complication	0 (0.0)	0 (0.0)	Any Complications	0 (0.00–0.00)
Cardiac pacing wires	*N*	2	2	Audit days	4
	Device failure	NA	0 (0.0)	Device Failure	0 (0.00–0.00)
	Any complication	0 (0.0)	0 (0.0)	Any Complications	0 (0.00–0.00)
Subcutaneous infusion	*N*	8	8	Audit days	25
	Device failure	NA	2 (25.0)	Device Failure	8.00 (2.00–31.99)
	Any complication	0 (0.0)	2 (25.0)	Any Complications	8.00 (2.00–31.99)
	Swelling	0 (0.0)	2 (25.0)	Swelling	
Cardiac lines	*N*	2	2	Audit days	4
	Device failure	0 (0.0)	0 (0.0)	Device Failure	0 (0.00–0.00)
	Any complications	0 (0.0)	0 (0.0)	Any Complications	0 (0.00–0.00)
ECMO	*N*	1	1	Audit days	4
	Device failure	NA	0 (0.0)	Device Failure	0 (0.00–0.00)
	Any complications	0 (0.0)	0 (0.0)	Any Complications	0 (0.00–0.00)
Ventricular assist device	*N*	1	1	Audit days	4
	Device failure	NA	0 (0.0)	Device Failure	0 (0.00–0.00)
	Any complications	0 (0.0)	1 (100.0)	Any Complications	25.00 (3.52–177.48)
	Clots in circuit	0 (0.0)	1 (100.0)	Clots in circuit	25.00 (3.52–177.48)

*CI* confidence interval, *ECMO* extracorporeal membrane oxygenation, *N* number, *NA* not applicable, *VAP* ventilator-associated pneumonia.

^a^Unable to assess is not counted as aggregated complication or failure.

On Day 0 of the audit, no cases of CLABSI were recorded. However, during the follow–up period, three instances of suspected CLABSIs were noted, none of which were confirmed upon further investigation. A single suspected local infection was noted on the Day 0 of the audit, with the number escalating to six during the subsequent follow-up. Again, none of these cases were ultimately confirmed as infections. The audit revealed one confirmed case of catheter-associated thrombosis at the outset. Despite the complication, the catheter did not need to be removed during the audit period. Two stage 1 pressure injuries related to vascular access devices were observed on Day 0, one of which progressed to stage 2 by the third day of follow-up.

On Day 0 of the audit, there were four skin injuries attributed to devices and dressings in the gastrointestinal devices (*n* = 112), a figure that doubled during the follow-up (*n* = 11). Additionally, two stage 1 pressure injuries associated with gastrointestinal devices were noted. Within the respiratory devices (*n* = 16), there was one confirmed case of ventilator-acquired pneumonia. For drains (*n* = 25), the most frequently reported complications on Day 0 were leakage (*n* = 3) and occlusion or poor drainage (*n* =2). During the follow-up, one suspected local infection in the drains was confirmed. For urinary devices, an occlusion was identified on Day 0 of the audit. Subsequent follow-up revealed instances of dislodgment, skin injuries related to devices and dressings, as well as damage to catheters.

There were no complications identified in epidural, regional/local infusion devices, cardiac pacing wires, and extracorporeal membrane oxygenation.

### Insertion and management documentation

Around 85% (*n* = 351) of devices had some documentation surrounding insertion, with the insertion date being known for 95% of devices (*n* = 392) (Table 4; Supplementary Table 11). On Day 0, 96% (*n* = 394) of the devices had been documented by nurses and 54% (*n* = 222) by medical staff in the previous 24 hours (Table 4; Supplementary Table 12). Over the entire audit duration (Days 0–3), 99% (*n* = 407) devices were documented at some point by nursing staff, and 75% (*n* = 310) devices were documented by medical staff.

**Table 4:** Dressing and pain assessments and documentations on day 0 and throughout the audit period (*N* = 412 devices).

	Vascular access devices	Gastrointestinal devices	Respiratory/airway	Drains	Urinary	Epidural infusion	Regional/local infusion	Cardiac pacing wires	Cardiac Lines	Subcutaneous infusion	ECMO	Ventricular assist device	Total
	*N* = 223	*N* = 112	*N* = 16	*N* = 25	*N* = 20	*N* = 1	*N* = 1	*N* = 2	*N* = 2	*N* = 8	*N* = 1	*N* = 1	*N* = 412
	*N*(%)	*N*(%)	*N*(%)	*N*(%)	*N*(%)	*N*(%)	*N*(%)	*N*(%)	*N*(%)	*N*(%)	*N*(%)	*N*(%)	*N*(%)
Insertion documentation													
Insertion documentation	188 (84.3)	93 (83.0)	15 (93.8)	25 (100.0)	18 (90.0)	0 (0.0)	1 (100.0)	2 (100.0)	2 (100.0)	5 (62.5)	1 (100.0)	1 (100.0)	351 (85.2)
Insertion date known	214 (95.9)	104 (92.8)	16 (100.0)	25 (100.0)	19 (95.0)	1 (100.0)	1 (100.0)	2 (100.0)	2 (100.0)	6 (75.0)	1 (100.0)	1 (100.0)	392 (95.2)
Documentation in past 24 h (on day 0)													
Nursing documentation	215 (96.4)	107 (95.5)	15 (93.8)	25 (100.0)	20 (100.0)	1 (100.0)	1 (100.0)	0 (0.0)	2 (100.0)	6 (75.0)	1 (100.0)	1 (100.0)	394 (95.6)
Medical documentation	118 (52.9)	56 (50.0)	12 (75.0)	19 (76.0)	9 (45.0)	1 (100.0)	1 (100.0)	0 (0.0)	1 (50.0)	3 (37.5)	1 (100.0)	1 (100.0)	222 (53.9)
Documentation in past 24 h (ever)													
Nursing documentation	221 (99.1)	110 (98.2)	16 (100.0)	25 (100.0)	20 (100.0)	1 (100.0)	1 (100.0)	2 (100.0)	2 (100.0)	7 (87.5)	1 (100.0)	1 (100.0)	407 (98.8)
Medical documentation	164 (73.5)	82 (73.2)	16 (100.0)	20 (80.0)	15 (75.0)	1 (100.0)	1 (100.0)	2 (100.0)	2 (100.0)	5 (62.5)	1 (100.0)	1 (100.0)	310 (75.2)
Dressing integrity (on day 0)													
Clean, dry, and intact	188 (84.3)	90 (80.4)	12 (75.0)	10 (40.0)	14 (70.0)	1 (100.0)	0 (0.0)	0 (0.0)	0 (0.0)	7 (87.5)	0 (0.0)	1 (100.0)	323 (78.4)
Not visible	12 (5.4)	4 (3.6)	1 (6.3)	6 (24.0)	2 (10.0)	0 (0.0)	1 (100.0)	2 (100.0)	1 (14.3)	0 (0.0)	0 (0.0)	0 (0.0)	29 (7.0)
Dressing integrity (ever)													
Clean, dry, and intact	171 (76.7)	73 (65.2)	10 (62.5)	14 (56.0)	19 (95.0)	1 (100.0)	1 (100.0)	1 (100.0)	1 (50.0)	8 (100.0)	1 (100.0)	1 (100.0)	302 (73.3)
Not visible	19 (8.6)	9 (8.2)	2 (12.5)	10 (40.0)	4 (20.0)	0 (0.0)	1 (100.0)	2 (100.0)	2 (28.6)	0 (0.0)	0 (0.0)	0 (0.0)	49 (11.9)
Device related pain (ever)													
Pain on day 0	23 (10.3)	6 (5.4)	0 (0.0)	2 (8.0)	3 (15.0)	0 (0.0)	0 (0.0)	0 (0.0)	0 (0.0)	2 (25.0)	0 (0.0)	0 (0.0)	36 (8.7)
Pain (ever)	39 (17.5)	10 (8.9)	1 (6.3)	11 (44.0)	3 (15.0)	0 (0.0)	0 (0.0)	0 (0.0)	1 (50.0)	2 (25.0)	1 (100.0)	1 (100.0)	69 (15.8)

*ECMO* extracorporeal membrane oxygenation.

### Dressing integrity

Around 78% (*n* = 323) of invasive devices had a dressing that was clean, dry, and intact, and 7% (*n* = 29) had a dressing that was reported as not visible on Day 0 (Table 4; Supplementary Table 13). Across the entire audit duration (Days 0–3), 73% (*n* = 302) of devices were reported to have dressings that were clean, dry and intact, and 12% (*n* = 49) had dressings that were not visible.

### Pain assessment

Around 9% (*n* = 36) of the devices were associated with pain on Day 0, with 16% (*n* = 69) of devices associated with pain across the entire audit duration (Days 0–3). Drains (*n* = 11; 44.0%), cardiac lines (*n* = 1; 50%), extracorporeal membrane oxygenation (*n* = 1; 100%), and ventricular assist device (*n* = 1; 100%) had high proportion of pain reported during the audit period (Table 4; Supplementary Table 14).

### Skin complications

Details of skin complications during the audit period are reported in Supplementary Table 15. In total, eight vascular access devices, 11 gastrointestinal devices, and one urinary device had skin complications during the audit period. On Day 0, skin tear (*n* = 1; 0.4%), irritant contact dermatitis (*n* = 3; 1.3%), and pressure injuries (2; 0.9%; both Stage 1) were reported in vascular access devices and irritant contact dermatitis (*n* = 4; 3.6%) and pressure injuries (*n* = 2; 1.8; both Stage 1) were reported in gastrointestinal devices. Throughout the audit, one of the pressure injuries in vascular access devices developed to Stage 2 on Day 3.

## Discussion

This is the first point and period prevalence study to demonstrate the current state of invasive devices’ utility and complications conducted globally, and was carried out across three independent, tertiary pediatric hospitals in Australia. The findings of this study provide valuable insights into the utility, complications, and documentation practices associated with invasive devices in pediatric patients. The audit revealed that the majority of patients had at least one device, with vascular access devices being the most prevalent. Device utility rates varied across different settings, with surgical and critical care wards having the highest rates for vascular access and other types of devices. These common, valuable devices cross boundaries of clinical practice, and represent key moments in healthcare experience for children.^18^ Device management also highlighted the use of a new type of long peripheral intravenous catheter emerging in clinical settings, which both were used in critical care settings. There was also a considerable proportion of peripheral vascular access devices still being inserted into the antecubital fossa, which is not recommended in the Infusion Nurses Society Standards of Practice (INSSoP)^19^ and by the Australian Commission on Safety and Quality in Health Care guideline.^20^

The overall point prevalence of device complications was 10.7% (*n* = 44), with vascular access devices (*n* = 30; 13.5% complications) and drains (*n* = 5; 20.0% complications) exhibiting the highest complication rates. However, no benchmarks are available that incorporate all of these devices in in-patient settings to compare the results. In an outpatient population, a retrospective study on device-complicated encounters in the emergency department reported that complications of 3 devices (central venous catheters, enteral tubes, and tracheostomy tubes) accounted for 13.0% of overall hospitalizations and 28% of overall emergency department visits.^21^ Central venous access device presence was associated with device-complicated emergency department visits, and gastrojejunostomy/jejunostomy tube presence (adjusted odds ratio 3.3 [95% CI 1.5–7.5]) was associated with device-complicated hospitalization.^21^ The finding highlights the importance of device management in general, as mismanagement of these devices can lead to unwanted hospitalizations in non-acute outpatients. This risk is further heightened within in-patient settings. Therefore, prioritizing appropriate device management practices is essential to minimize complications and prevent unnecessary hospitalizations in both outpatient and inpatient populations.

The high prevalence of complications, coupled with the commonality of vascular access devices, highlights the importance of management measures and ongoing surveillance to minimize the risk of all complications. These strategies need to be tailored to the type of vascular access device—as the types of complications experienced differ greatly. In a cross-sectional study of 4206 peripheral intravenous catheters, 11.4% of these devices (*n* = 479) had complications, with pain and tenderness on palpation being the most frequently reported (*n* = 209) followed by blood in the line (*n* = 71).^22^ In the meta-analysis of 32 observational studies, the pooled proportion of peripheral intravenous catheter failure was 38% (95% CI: 0.32–0.45) by device, with infiltration being the most common cause for failure (10%; 95% CI: 0.07–0.14), followed by accidental removal, occlusion, and leakage.^23^ In a systematic review of complications of central venous access devices, the authors reported that 25% (95% CI: 21–29%) of central venous access devices failed before the completion of therapy and concluded that central venous access device failure and complications in pediatrics are a significant burden on the health care system internationally. Our point prevalence of any complications for all vascular access devices was 13.5% (*n* = 30), and the period prevalence of device failure was 16.1% (*n* = 36), which was lesser than the pooled estimates in meta-analyses. Additionally, our study had no confirmed CLABSI and catheter-associated local infections, which was lower than previous systematic reviews and individual studies.^2,24^ We also had lower device and dressing-related skin injury than the previous study,^25^ but we had a similar proportion of dermatitis and skin tear in the secondary analysis involving 10,859 catheters.^26^ Our pressure injury period prevalence was 1.3% (*n* = 3) for central venous access devices and 1.8% (*n* = 2) for gastrointestinal devices. This equates to 1.2% of the prevalence of all devices, which is lower than the pooled prevalence of medical device-related pressure injuries reported in a systematic review of 25,742 pediatric patients (7%; 95% CI 5.5–8.8%) in the United States.^27^ The overall lower prevalence of complications could be from the short follow-up period due to the study design and the timing of the audit. Given that more than half of the devices in our study are vascular access devices, including central venous access devices, this area continues to be deserving of further research and improved management protocols to reduce complications related to these widely-used devices.

In our study, the condition of the dressing remained clean, dry, and undamaged for 78.0% of the devices on the first day and 73.0% throughout the evaluation period. There is no universally accepted standard for all device dressings, but an 80.0% preservation of dressing integrity was the objective of a peripheral intravenous catheter quality improvement project,^28^ which is similar to our study. However, up to 12.0% of dressing sites were not visible during the audit period, which may have impacted our ability to assess for complications fully. Our study found that dressings were more likely to be intact on devices requiring frequent observations, such as vascular access devices and ventricular assist devices, compared to others, indicating that clinical priorities influenced adherence to dressing protocols. Dressing integrity is connected with dressing-related skin injury, catheter-related infections, and dislodgment.^29^ While device and dressing-related skin injury and dislodgment are common complications in vascular access devices, gastrointestinal devices, and urinary devices, there is a need for more research on dressing and securement and enhanced attention to dressing care and maintenance to prevent potential complications.

The study revealed that documentation practices were generally favorable, with a majority of devices having insertion documented, known insertion dates, and device documentation. This is possible because two of the hospitals had electronic medical records and facilitated the documentation completion.^30^ However, it was observed that any type of documentation was more likely to occur for devices supporting the provision of advanced physiological supports, such as extracorporeal membrane oxygenation, ventricular assist device, and pacing wires, rather than for devices like subcutaneous infusions, drains and urinary devices. In our project, we used a binary report (yes/no) of pain associated with the device, to avoid the use of multiple pain scales that may challenge the auditing process due to differences in practice across different staff and clinical environments. There were also up to 14.0% of cases where pain was unable to be assessed, which may have contributed to the potential underreporting of device-associated pain.

The study’s strengths include a rigorous prospective data collection process and used a multi-site approach. However, there are limitations to consider. The study was conducted in a tertiary healthcare setting, which may limit the generalizability of the findings to other contexts. Additionally, the study focused on point prevalence rather than longitudinal follow-up, which may impact the understanding of device-related complications over time. It is worth noting that the timing of the audit of each site was determined based on staff logistics and feasibility, which limited the ability to conduct the audit during periods of high patient acuity. Therefore, our results may not fully capture the challenges and complexities associated with invasive device management during high-acuity situations. During acute situations, such as staffing shortages, disease outbreaks or high patient volumes, healthcare providers may face additional challenges in device utilization, management, and documentation.^31^ The increased workload, time constraints, and competing priorities may impact the adherence to best practices and protocols related to invasive devices.^32^ Therefore, the findings of this study may not fully represent the prevalence of some devices, complications and documentation practices. Despite the limitations associated with the timing of the audit, the findings of this study still contribute valuable information on device utility, complications, and documentation practices in a pediatric healthcare setting.

## Conclusion

Invasive devices are essential for the management of pediatric patients, but they come with inherent risks. This study examined the utilization and complications of invasive devices in three independent tertiary pediatric hospitals in Australia. The findings shed light on the current state of these devices, their associated complications, and documentation practices in pediatric patients. The audit revealed a high prevalence of invasive devices, particularly vascular access devices. Device utilization rates varied across different hospital settings, with surgical and critical care wards demonstrating the highest rates of both vascular access devices and other types of devices. These insights provide valuable information for improving the use, management, and monitoring of invasive devices in pediatric healthcare.

### Supplementary information

Supplementary Information

## Data Availability

De-identified participant data, the data dictionary, and related documents (e.g., case report forms) will be made available on written request to the senior author. Requests must be accompanied by a formal protocol for the use of the data and approval from the relevant Human Research Ethics Committees. A written and signed data access agreement will be required.
