# Supplementary material for: Pediatric invasive device utility and harm: a multi-site point prevalence survey
Source: Pediatr Res. 2024 Jan 11;96(1):148–58. doi: 10.1038/s41390-023-03014-1 (PMC11257939; doi:10.1038/s41390-023-03014-1)
Supplement: Supplementary file 1 — Supplementary Information [file 41390_2023_3014_MOESM1_ESM.pdf]

Supplementary Table 1: Inter-rater reliability of auditors

		**Site A**	**Site B**	**Site C**	**Total**
		Coefficient (95% CI)	Coefficient (95% CI)	Coefficient (95% CI)	Coefficient (95% CI)
**Demographics**					
	Percent agreement	0.83 (0.56-1.00)	Complete agreement	0.83 (0.56-1.00)	0.77 (0.49-1.00)
	Kappa	0.04 (-0.11-0.20)	Complete agreement	0.26 (-0.04-0.56)	0.29 (-0.11-0.69)
	Gwet's AC	0.82 (0.49-1.00)	Complete agreement	0.81 (0.47-1.00)	0.74 (0.39-1.00)
**Devices**	Percent agreement	0.92 (0.76-1.00)	0.95 (0.85-1.00)	0.94 (0.80-1.00)	0.88 (0.70-1.00)
	Kappa	0.13 (0.07-0.21)	-0.02 (-0.08-0.03)	0.30 (0.22-0.38)	0.28 (0.21-0.36)
	Gwet's AC	0.92 (0.74-1.00)	0.95 (0.84-1.00)	0.93 (0.77-1.00)	0.87 (0.65-1.00)

CI: confidence interval; Scale: <0.00 Poor, 0.00-0.20 Slight, 0.20-0.40 Fair, 0.40-0.60 Moderate, 0.60-0.80 Substantial, 0.80-1.00 Almost perfect (Landis, J. R., and G. G. Koch. 1977a. The measurement of observer agreement for categorical data. Biometrics 33:159–174)

Supplementary Table 2: Devices types at day 0 (N=412)

Device type	N (% of total number of devices)	Types	N (% of total number of devices)	Subtypes	N (%of total number of devices)
Vascular access devices	223 (54.1)	Peripheral intravenous venous catheter	114 (27.7)		
		Midline	7 (1.6)		
		Arterial line	8 (1.9)		
		Central venous access device	94 (22.8)	Peripherally inserted central catheter	29 (7.0)
				Implanted port	13 (3.2)
				Tunneled cuffed	30 (7.3)
				Tunneled non-cuffed	4 (1.0)
				Non tunneled	14 (3.4)
				Hemodialysis	2 (0.5)
				Umbilical	2 (0.5)
Gastrointestinal devices	112 (27.2)	Nasogastric/ transpyloric	89 (21.6)	Gastric	83 (20.1)
				Transpyloric	6 (1.5)
		Orogastric tube	4 (1.0)		
		Percutaneous Endoscopic Tube	17 (4.1)	Percutaneous endoscopic gastrostomy device	7 (1.7)
				Low profile (Mickey button)	9 (2.2)
				Long tube balloon	1 (0.2)
		Replogle	1 (0.2)		
		Cecostomy (with the drain tube)	1 (0.2)		
Drains	25 (6.1)	Peritoneal dialysis	2 (0.5)		
		Wound surgical drain	6 (1.5)		
		External ventricular drain	1 (0.2)		
		Chest drains	16 (3.9)		
Urinary device	20 (4.9)	Urinary catheter	19 (4.6)		
		Mitrofanoff	1 (0.2)		
Respiratory/ Airway device	16 (3.9)	Endotracheal	10 (2.4)		
		Tracheostomy tube	4 (1.0)		
		Nasopharyngeal stent	2 (0.5)		
Subcutaneous	8 (1.9)	Insuflons	5 (1.2)		
		Continuous glucose monitoring	3 (0.6)		
Cardiac lines	2 (0.5)	Direct left atrial lines			
Cardiac pacing wires	2 (0.5)	Epicardial			
Epidural infusion	1 (0.2)				
Regional/ local infusion	1 (0.2)	Pump			
Extracorporeal membrane oxygenation	1 (0.2)	Veno-venous			
Ventricular assist device	1 (0.2)	Left ventricular assist device			

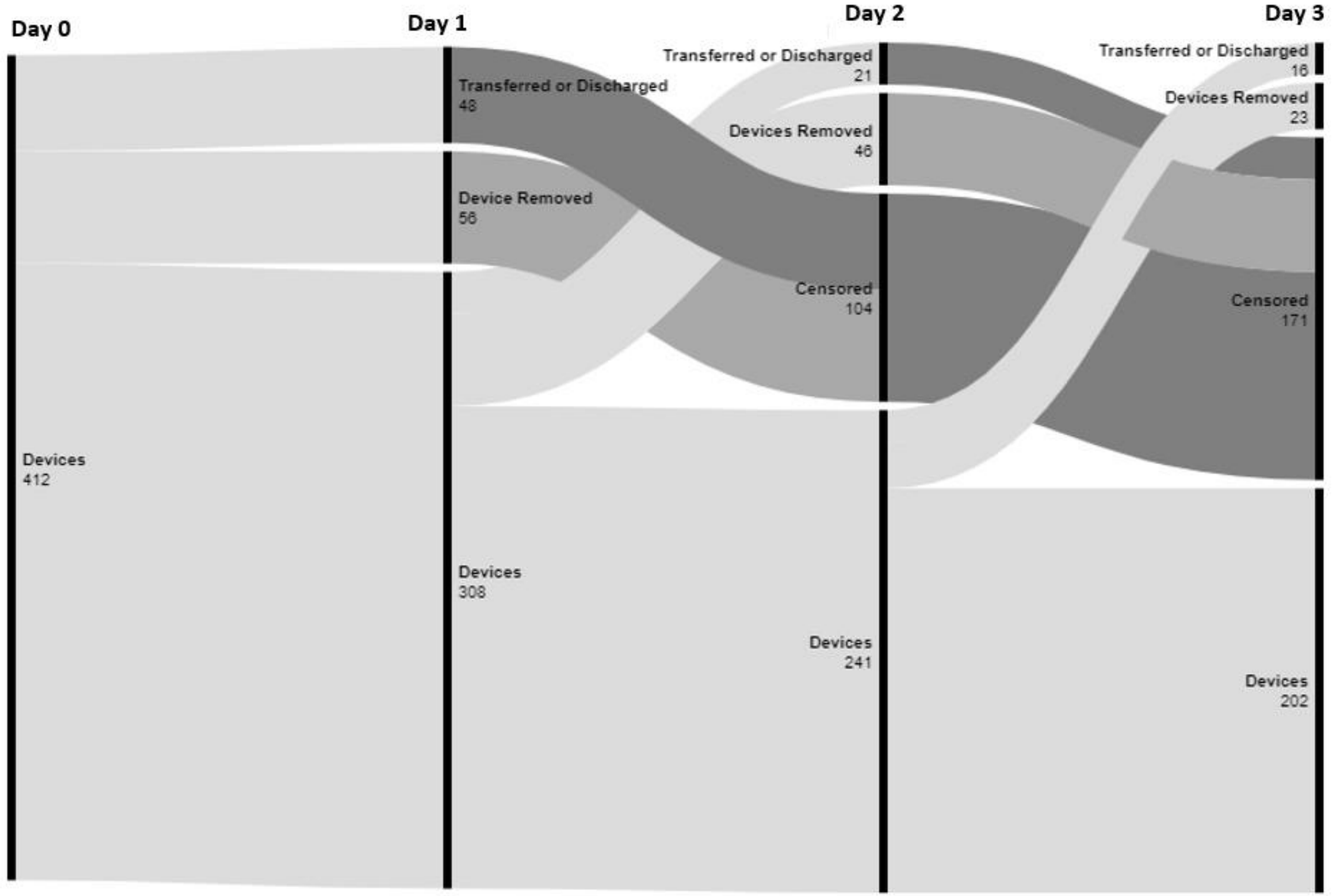

Supplementary Figure 1: Flow of devices during the audit

Supplementary Table 3: Device characteristics of peripheral vascular access devices (N=129)

		PIVC (N=114) N (%)	Midline (N=7) N (%)	Arterial line (N=8) N (%)
**Location**				
	Hand	44 (38.6)	0 (0.0)	0 (0.0)
	Wrist	6 (5.3)	1 (14.3)	4 (50.0)
	Upper arm	0 (0.0)	0 (0.0)	1 (12.5)
	Forearm	36 (31.6)	4 (57.1)	1 (12.5)
	Antecubital fossa	21 (18.4)	1 (14.3)	1 (12.5)
	Groin	0 (0.0)	0 (0.0)	1 (12.5)
	Lower leg	0 (0.0)	1 (14.3)	0 (0.0)
	Foot ankle	7 (6.1)	0 (0.0)	0 (0.0)
**Side**				
	Left	52 (45.6)	5 (71.4)	2 (25.0)
	Right	62 (54.4)	2 (28.6)	6 (75.0)
**Size**				
	16g	3 (2.6)	0 (0.0)	0 (0.0)
	18g	5 (4.4)	0 (0.0)	0 (0.0)
	20g	12 (10.5)	0 (0.0)	0 (0.0)
	22g	47 (41.2)	4 (44.4)	5 (62.5)
	22g Long PIVC	4 (3.5)	0 (0.0)	0 (0.0)
	22g Integrated	2 (1.8)	0 (0.0)	0 (0.0)
	24g	18 (15.8)	1 (11.1)	2 (25.0)
	3fr	0 (0.0)	1 (11.1)	0 (0.0)
	Unknown	23 (20.2)	1 (11.1)	1 (12.5)

Abbreviations: PIVC: Peripheral intravenous catheter

Supplementary Table 4: Device characteristics of central venous access devices (N= 94)

		PICC (N=29) N (%)	Port (N=13) N (%)	Tunneled cuffed (N=30) N (%)	Tunneled non-cuffed (N=4) N (%)	Non-tunneled (N=14) N (%)	HD (N=2) N (%)	Umbilical (N=2) N (%)
**Side**								
	Left	10 (34.5)	3 (23.1)	8 (26.7)	2 (50.0)	3 (21.4)	NA	NA
	Right	19 (65.5)	10 (76.9)	22 (73.3)	2 (50.0)	11 (78.6)	NA	NA
**Size (fr)**								
	2.0-2.9	3 (10.3)	0 (0.0)	0 (0.0)	0 (0.0)	1 (7.1)	0 (0.0)	0 (0.0)
	3.0-3.9	15 (51.7)	0 (0.0)	0 (0.0)	1 (25.0)	1 (7.1)	0 (0.0)	1 (50.0)
	4-4.9	4 (13.8)	0 (0.0)	0 (0.0)	2 (50.0)	10 (71.4)	0 (0.0)	0 (0.0)
	5-5.9	3 (10.3)	0 (0.0)	4 (13.3)	1 (25.0)	1 (7.1)	0 (0.0)	1 (50.0)
	6-6.9	0 (0.0)	6 (46.2)	1 (3.3)	0 (0.0)	1 (7.1)	0 (0.0)	0 (0.0)
	7-7.9	0 (0.0)	3 (23.1)	22 (73.3)	0 (0.0)	0 (0.0)	0 (0.0)	0 (0.0)
	8-8.9	0 (0.0)	0 (0.0)	0 (0.0)	0 (0.0)	0 (0.0)	1 (50.0)	0 (0.0)
	9-9.9	0 (0.0)	0 (0.0)	1 (3.3)	0 (0.0)	0 (0.0)	0 (0.0)	0 (0.0)
	Other	0 (0.0)	0 (0.0)	1 (3.3; size 10)	0 (0.0)	0 (0.0)	1 (50.0; size 12)	0 (0.0)
	Unknown	4 (13.8)	4 (30.8)	1 (3.3)	0 (0.0)	0 (0.0)	0 (0.0)	0 (0.0)
**Number of lumens**	1	17 (58.6)	10 (76.9)	1 (3.3)	2 (50.0)	1 (7.1)	NA	NA
	2	11 (37.9)	3 (23.1)	29 (96.7)	2 (50.0)	0 (0.0)	NA	NA
	3	1 (3.4)	0 (0.0)	0 (0.0)	0 (0.0)	13 (92.9)	NA	NA

Abbreviations: HD: Hemodialysis; PICC: Peripherally inserted central catheter; NA: not applicable.

Supplementary Table 5: Device characteristics of gastrointestinal devices (N=112)

		Nasogastric/ transpyloric (N=89) N (%)	Orogastric tube (N=4) N (%)	Percutaneous endoscopic gastrostomy device (N=7) N (%)	Low profile (Mickey button) (N=9) N (%)	Long tube balloon (N=1) N (%)	Replogle (N=1) N (%)	Cecostomy with the drain tube (N=1) N (%)
**Type**	Gastric	83 (93.3)	NA	NA	NA	NA	NA	NA
	Transpyloric	6 (6.7)	NA	NA	NA	NA	NA	NA
**Side**	Right nare	43 (48.3)	NA	NA	NA	NA	NA	NA
	Left nare	46 (51.7)	NA	NA	NA	NA	NA	NA
	Right side of mouth	0 (0.0)	4 (100.0)	NA	NA	NA	NA	NA
	Stomach	0 (0.0)	0 (0.0)	NA	NA	NA	NA	NA
	Jejunal/small intestine	0 (0.0)	0 (0.0)	NA	NA	NA	NA	NA
**Size**	6fr	32 (36.0)	3 (75.0)	0 (0.0)	NA	NA	NA	NA
	8fr	37 (41.6)	1 (25.0)	0 (0.0)	NA	NA	NA	NA
	10fr	13 (14.6)	0 (0.0)	0 (0.0)	NA	NA	NA	NA
	12fr	3 (3.4)	0 (0.0)	1 (14.3)	1 (11.1)	0 (0.0)	NA	NA
	14fr	1 (1.1)	0 (0.0)	2 (28.6)	3 (33.3)	1 (100.0)	NA	NA
	16fr	0 (0.0)	0 (0.0)	1 (14.3)	1 (11.1)	0 (0.0)	NA	NA
	18fr	0 (0.0)	0 (0.0)	0 (0.0)	3 (33.3)	0 (0.0)	NA	NA
	Other	0 (0.0)	0 (0.0)	1 (14.3)	1 (11.1)	0 (0.0)	NA	NA
	unknown	3 (3.4)	0 (0.0)	2 (28.6)	0 (0.0)	0 (0.0)	NA	NA

Abbreviations: NA: not applicable.

Supplementary Table 6: Device characteristics of respiratory devices (N=16)

		Endotracheal<br>(N=10)<br>N (%)	Tracheostomy tubes<br>(N=4)<br>N (%)	Nasopharyngeal stent<br>(N=2)<br>N (%)
**Side**	L) lips	1 (10.0)	NA	2 (100.0)
	L) nare	2 (20.0)	NA	0 (0.0)
	R) Lips	1 (10.0)	NA	0 (0.0)
	R) nare	6 (60.0)	NA	0 (0.0)
**Size**				
	3-3.9 mm	8 (80.0)	0 (0.0)	0 (0.0)
	4-4.9mm	2 (20.0)	0 (0.0)	0 (0.0)
	3.5 fr	0 (0.0)	1 (25.0)	1 (50.0)
	4.0 fr	0 (0.0)	2 (50.0)	0 (0.0)
	7.0 fr	0 (0.0)	1 (25.0)	1 (50.0)
**Cuffed**		7 (70.0)	0 (0.0)	NA
**Uncuffed**		3 (30.0)	4 (100.0)	NA
**Inner cannula present**		NA	1 (25.0)	NA
**Long cannula**		NA	1 (25.0)	NA
**Pediatric cannula**		NA	1 (25.0)	NA

Abbreviations: NA: not applicable

Supplementary Table 7: Device characteristics of drain devices (N=25)

		Wound surgical drain (N=6) N (%)	Chest drain (N=16) N (%)	Peritoneal dialysis (N=2) N (%)	EVD standard indication (N=1) N (%)
**Size**	8-9 fr	NA	2 (12.5)	0 (0.0)	NA
	10 fr	NA	7 (43.8)	0 (0.0)	NA
	14 fr	NA	3 (18.8)		NA
	15 fr	NA	1 (6.3)	1 (50.0)	NA
	37cm double cuffed	NA	0 (0.0)	1 (50.0)	NA
	pigtail	NA	1 (6.3)	0 (0.0)	NA
	unknown	NA	2 (12.5)	0 (0.0)	NA
**Suction**	Passive	1 (16.6)	NA	NA	NA
	Active	5 (83.3)	NA	NA	NA
**Location**	Chest or back	2 (33.3)	16 (100.0)	NA	NA
	Head or neck	2 (33.3)	NA	NA	1 (100.0)
	Abdo or pelvis	2 (33.3)	NA	2 (100.0)	NA
**ICC type**	Upper ICC	NA	5 (83.3)	NA	NA
	Lower ICC	NA	1 (16.6)	NA	NA
**Side**	Left	2 (33.3)	3 (50.0)	NA	NA
	Right	4 (66.6)	3 (50.0)	NA	NA
**Indication for EVD**	Standard	NA	NA	NA	1 (100.0)

Abbreviations: EVD: External ventricular drain; ICC: intercoastal catheters; NA: Not Applicable

Supplementary Table 8: Device characteristics of urinary devices (N=20)

	N (%)	Size	N (%)
**IDC**	19 (95.0)	6fr	9 (47.3)
		8fr	4 (21.1)
		10fr	3 (15.8)
		12fr	1 (5.3)
		18fr	1 (5.3)
		Unknown	2 (10.5)
**Mitraoffnanoff**	1 (5.0)	NA	NA

Abbreviations: IDC: Indwelling urinary catheter; NA: not applicable

Supplementary Table 9: Device characteristics of epidural, regional and subcutaneous lines (n=10)

			N (%)
**Epidural (N=1)**	**Size**	16 g	1 (100.0)
	**Location**	Lumbar	1 (100.0)
**Regional (N=1)**	**Type**	Pump	1 (100.0)
	**Location**	Femur, knee, lower leg	1 (100.0)
**Subcutaneous (N=8)**	**Type**	Insuflon	5 (62.5)
		Continuous glucose monitoring	3 (37.5)
	**Size**	25g	1 (12.5)
		27g	3 (37.5)
		Unknown	4 (50.0)
	**Location**	Thigh	5 (62.5)
		Unknown	3 (37.5)

Supplementary Table 10: Device characteristics of pacing wires, cardiac lines, ventricular assist devices and extracorporeal membrane oxygenation (N=5)

	Type	N (%)	Location	N (%)
**Epicardial (N=2)**	Ventricular	1 (50.0)	Chest	1 (50.0)
	Atrial	1 (50.0)	Chest	1 (50.0)
**Cardiac lines (N=2)**	Direct left atrial lines	2 (100.0)	Chest	2 (100.0)
**VAD (N=1)**	LVAD Berlin Heart	1 (100.0)	Unknown	1 (100.0)
**ECMO (N=1)**	V-V (13 fr)	1 (100.0)	Neck	1 (100.0)

Abbreviations: ECMO: extracorporeal membrane oxygenation; LVAD: left ventricular assist device; VAD: ventricular assist device; V-V: veno-venous

Supplementary Table 11: Insertion documentation and insertion date known (N=412)

	Insertion Documentation		Insertion date known			Total
	N (%)		N (%)			N (%)
	Yes	No	Yes	No	Unknown	
**Vascular access devices**	188 (84.3)	35 (15.7)	214 (96.0)	2 (0.9)	7 (3.1)	223 (100.0)
**Gastrointestinal devices**	93 (83.0)	19 (16.9)	104 (92.9)	1 (0.9)	7 (6.3)	112 (100.0)
**Respiratory/ airway**	15 (93.8)	1 (6.3)	16 (100.0)	0 (0.0)	0 (0.0)	16 (100.0)
**Drains**	25 (100.0)	0 (0.0)	25 (100.0)	0 (0.0)	0 (0.0)	25 (100.0)
**Urinary**	18 (90.0)	2 (2.0)	19 (95.0)	0 (0.0)	1 (5.0)	20 (100.0)
**Epidural infusion**	0 (0.0)	1 (100.0)	1 (100.0)	0 (0.0)	0 (0.0)	1 (100.0)
**Regional/ local infusion**	1 (100.0)	0 (0.0)	1 (100.0)	0 (0.0)	0 (0.0)	1 (100.0)
**Cardiac pacing wires**	2 (100.0)	0 (0.0)	2 (100.0)	0 (0.0)	0 (0.0)	2 (100.0)
**Cardiac Lines**	2 (100.0)	0 (0.0)	2 (100.0)	0 (0.0)	0 (0.0)	2 (100.0)
**Subcutaneous infusion**	5 (62.5)	3 (37.5)	6 (75.0)	0 (0.0)	2 (25.0)	8 (100.0)
**ECMO**	1 (100.0)	0 (0.0)	1 (100.0)	0 (0.0)	0 (0.0)	1 (100.0)
**Ventricular assist device**	1 (100.0)	0 (0.0)	1 (100.0)	0 (0.0)	0 (0.0)	1 (100.0)

Abbreviations: ECMO: extracorporeal membrane oxygenation

Supplementary Table 12: Documentation during the audit period

	Vascular access devices	Gastrointestinal devices	Respiratory/ airway	Drains	Urinary	Epidural infusion	Regional/ local infusion	Cardiac pacing wires	Cardiac lines	Subcutaneous infusion	ECMO	Ventricular assist device	Total
	N (%)	N (%)	N (%)	N (%)	N (%)	N (%)	N (%)	N (%)	N (%)	N (%)	N (%)	N (%)	N (%)
**Day 0**	**N= 223**	**N= 112**	**N= 16**	**N= 25**	**N= 20**	**N= 1**	**N= 1**	**N= 2**	**N= 2**	**N= 8**	**N=1**	**N=1**	**N=412**
Nurse's documentation in past 24 hours	215 (96.4)	107 (95.5)	15 (93.8)	25 (100.0)	20 (100.0)	1(100.0)	1(100.0)	0 (0.0)	2 (100.0)	6 (75.0)	1 (100.0)	1 (100.0)	394 (95.6)
Doctor's documentation in past 24 hours	118 (52.9)	56 (50.0)	12 (75.0)	19 (76.0)	9 (45.0)	1(100.0)	1(100.0)	0 (0.0)	1 (50.0)	5 (62.5)	1 (100.0)	1 (100.0)	222 (53.9)
**Day 1**	**N= 184**	**N=105**	**N= 16**	**N= 23**	**N= 20**	**N= 1**	**N= 1**	**N= 2**	**N= 2**	**N= 8**	**N= 1**	**N= 1**	**N=364**
Nurse's documentation in past 24 hours	178 (96.7)	95 (90.5)	16 (100.0)	22 (95.7)	20 (100.0)	1 (100.0)	1(100.0)	2(100.0)	2 (100.0)	6 (75.0)	1 (100.0)	1 (100.0)	345 (94.8)
Doctor's documentation in past 24 hours	97 (52.7)	56 (53.3)	15 (93.8)	12 (52.2)	8 (40.0)	1 (100.0)	1 (100.0)	2 (100.0)	2 (100.0)	4 (50.0)	1 (100.0)	1 (100.0)	200 (54.9)
**Day 2**	**N= 138**	**N= 87**	**N= 13**	**N= 19**	**N= 14**	**N= 1**	**N= 1**	**N= 2**	**N= 2**	**N= 8**	**N= 1**	**N= 1**	**N= 287**
Nurse's documentation in past 24 hours	135	85 (97.7)	13 (100.0)	19 (100.0)	14 (100.0)	1 (100.0)	1 (100.0)	2 (100.0)	1 (50.0)	7 (87.5)	1(100.0)	1(100.0)	280 (97.6)
Doctor's documentation in past 24 hours	71	42 (48.3)	11 (84.6)	14 (73.7)	6 (42.9)	1 (100.0)	0 (0.0)	2 (100.0)	1 (50.0)	2 (25.0)	1(100.0)	1(100.0)	152 (53.0)
**Day 3**	**N= 105**	**N= 75**	**N= 11**	**N= 17**	**N= 8**	**N= 1**	**N=1**	**N=0**	**N=0**	**N=5**	**N=1**	**N=1**	**N=225**
Nurse's documentation in past 24 hours	103 (98.1)	71 (94.7)	11 (100.0)	16 (94.1)	8 (100.0)	1 (100.0)	1 (100.0)	-	-	4 (80.0)	1(100.0)	1(100.0)	217 (96.4)
Doctor's documentation in past 24 hours	44 (41.9)	30 (40.0)	10 (90.9)	8 (47.1)	1 (12.5)	1 (100.0)	1 (100.0)	-	-	1 (20.0)	1(100.0)	1(100.0)	98 (43.6)

Abbreviations: ECMO: extracorporeal membrane oxygenation

Supplementary Table 13: Dressing assessment throughout the audit period

	Vascular access devices	Gastroint estinal devices	Respirato ry/ airway	Drains	Urinary	Epidural infusion	Regional/ local infusion	Cardiac pacing wires	Cardiac Lines	Subcutane ous infusion	ECMO/ cardiac lines	Ventricul ar assist device	Total
	N (%)	N (%)	N (%)	N (%)	N (%)	N (%)	N (%)	N (%)	N (%)	N (%)	N (%)	N (%)	N (%)
**Day 0**	**N= 223**	**N= 112**	**N= 16**	**N= 25**	**N= 20**	**N= 1**	**N= 1**	**N= 2**	**N=2**	**N=8**	**N= 1**	**N= 1**	**N= 412**
**Not clean**	22 (9.9)	13 (11.6)	3 (18.8)	9 (36.0)	1 (5.0)	0 (0.0)	0 (0.0)	0 (0.0)	1 (50.0)	0 (0.0)	0 (0.0)	0 (0.0)	49 (11.9)
**Clean dry and intact**	188 (84.3)	90 (80.4)	12 (75.0)	10 (40.0)	14 (70.0)	1 (100.0)	0 (0.0)	0 (0.0)	0 (0.0)	7 (87.5)	0 (0.0)	1 (100.0)	323 (78.4)
**No dressing**	1 (0.4)	5 (4.5)	0 (0.0)	0 (0.0)	3 (15.0)	0 (0.0)	0 (0.0)	0 (0.0)	0 (0.0)	1 (12.5)	1 (100.0)	0 (0.0)	11 (2.7)
**Not visible**	12 (5.4)	4 (3.6)	1 (6.3)	6 (24.0)	2 (10.0)	0 (0.0)	1 (100.0)	2 (100.0)	1 (50.0)	0 (0.0)	0 (0.0)	0 (0.0)	29 (7.0)
**Day 1**	**N= 155**	**N= 91**	**N= 13**	**N= 19**	**N= 14**	**N= 1**	**N= 1**	**N= 2**	**N=2**	**N=8**	**N= 1**	**N= 1**	**N= 308**
**Not clean**	19 (12.3)	16 (17.6)	0 (0.0)	3 (15.8)	0 (0.0)	0 (0.0)	0 (0.0)	0 (0.0)	1 (50.0)	0 (0.0)	0 (0.0)	0 (0.0)	39 (12.7)
**Clean dry and intact**	132 (85.2)	70 (76.9)	12 (92.3)	12 (63.2)	7 (50.0)	1 (100.0)	1 (100.0)	2 (100.0)	0 (0.0)	7 (87.5)	0 (0.0)	1 (100.0)	245 (79.5)
**No dressing**	0 (0.0)	5 (5.5)	1 (7.7)	0 (0.0)	6 (42.9)	0 (0.0)	0 (0.0)	0 (0.0)	0 (0.0)	1 (12.5)	1 (100.0)	0 (0.0)	14 (4.5)
**Not visible**	4 (2.6)	0 (0.0)	0 (0.0)	4 (21.1)	1 (7.1)	0 (0.0)	0 (0.0)	0 (0.0)	1 (50.0)	0 (0.0)	0 (0.0)	0 (0.0)	10 (3.2)
**Day 2**	**N= 114**	**N= 81**	**N= 11**	**N= 17**	**N= 8**	**N= 1**	**N= 1**	**N =0**	**N=0**	**N=5**	**N= 1**	**N= 1**	**N= 240**
**Not clean**	13 (11.14)	17 (21.0)	1 (9.1)	2 (11.8)	0 (0.0)	0 (0.0)	0 (0.0)	NA	NA	0 (0.0)	0 (0.0)	0 (0.0)	33 (13.8)
**Clean dry and intact**	97 (85.1)	62 (76.5)	9 (81.8)	12 (70.6)	4 (50.0)	1 (100.0)	0 (0.0)	NA	NA	4 (80.0)	0 (0.0)	1 (100.0)	190 (79.2)
**No dressing**	0 (0.0)	1 (1.2)	1 (9.1)	0 (0.0)	3 (37.5)	0 (0.0)	0 (0.0)	NA	NA	1 (20.0)	1 (100.0)	0 (0.0)	7 (2.9)
**Not visible**	4 (3.5)	1 (1.2)	0 (0.0)	3 (17.6)	1 (12.5)	0 (0.0)	1 (100.0)	NA	NA	0 (0.0)	0 (0.0)	0 (0.0)	10 (4.2)
**Day 3**	N= 92	**N=73**	N= 10	N= 14	N= 6	N= 1	N=0	N=0	N=0	**N=4**	N= 1	N= 1	N= 202
**Not clean**	19 (20.7)	15 (20.6)	3 (30.0)	1 (7.1)	0 (0.0)	0 (0.0)	NA	NA	NA	0 (0.0)	0 (0.0)	0 (0.0)	38 (18.8)

**Clean dry and intact**	69 (75.0)	52 (71.2)	5 (50.0)	6 (42.9)	1 (16.7)	1 (100.0)	NA	NA	NA	3 (75.0)	0 (0.0)	1 (100.0)	138 (68.3)
**No dressing**	0 (0.0)	2 (2.7)	1 (10.0)	0 (0.0)	4 (66.7)	0 (0.0)	NA	NA	NA	0 (0.0)	1 (100.0)	0 (0.0)	8 (4.0)
**Not visible**	4 (4.3)	4 (5.5)	1 (10.0)	7 (50.0)	1 (16.7)	0 (0.0)	NA	NA	NA	1 (25.0)	0 (0.0)	0 (0.0)	18 (8.9)

Abbreviations: NA: Not Applicable

Supplementary Table 14: Pain assessments

	Vascular access devices	Gastrointestinal devices	Respiratory/ airway	Drains	Urinary	Epidural infusion	Regional/ local infusion	Cardiac pacing wires	Cardiac Lines	Subcutaneous infusion	ECMO/ cardiac lines	Ventricular assist device	Total
	N (%)	N (%)	N (%)	N (%)	N (%)	N (%)	N (%)	N (%)	N (%)	N (%)	N (%)	N (%)	N (%)
**Day 0**	**N= 223**	**N= 112**	**N= 16**	**N= 25**	**N= 20**	**N= 1**	**N= 1**	**N= 2**	**N= 2**	**N= 8**	**N= 1**	**N= 1**	**N= 412**
Patient reported	15 (6.7)	4 (3.6)	0 (0.0)	1 (4.0)	3 (15.0)	0 (0.0)	0 (0.0)	0 (0.0)	0 (0.0)	0 (0.0)	0 (0.0)	0 (0.0)	23 (5.6)
Parent reported	4 (1.8)	2 (1.8)	0 (0.0)	0 (0.0)	0 (0.0)	0 (0.0)	0 (0.0)	0 (0.0)	0 (0.0)	0 (0.0)	0 (0.0)	0 (0.0)	6 (1.5)
Clinician reported	4 (1.8)	0 (0.0)	0 (0.0)	1 (4.0)	0 (0.0)	0 (0.0)	0 (0.0)	0 (0.0)	0 (0.0)	2 (25.0)	0 (0.0)	0 (0.0)	7 (1.7)
No pain	194 (87.0)	100 (89.3)	15 (93.8)	21 (84.0)	17 (85.0)	0 (0.0)	1 (100.0)	2 (100.0)	2 (100.0)	5 (62.5)	1 (100.0)	1 (100.0)	359 (87.1)
Unable to assess	6 (2.7)	6 (5.4)	1 (6.3)	2 (8.0)	0 (0.0)	1 (100.0)	0 (0.0)	0 (0.0)	0 (0.0)	0 (0.0)	0 (0.0)	0 (0.0)	17 (4.1)
**Day 1**	**N= 155**	**N= 91**	**N= 13**	**N= 19**	**N= 14**	**N= 1**	**N= 1**	**N= 2**	**N= 2**	**N= 8**	**N= 1**	**N= 1**	**N= 308**
Patient reported	3 (1.9)	4 (4.4)	0 (0.0)	1 (5.3)	1 (7.1)	0 (0.0)	0 (0.0)	0 (0.0)	0 (0.0)	0 (0.0)	0 (0.0)	0 (0.0)	9 (2.9)
Parent reported	0 (0.0)	1 (1.1)	0 (0.0)	0 (0.0)	0 (0.0)	0 (0.0)	0 (0.0)	0 (0.0)	0 (0.0)	0 (0.0)	0 (0.0)	0 (0.0)	1 (0.3)
Clinician reported	4 (2.6)	0 (0.0)	1 (7.7)	6 (31.6)	0 (0.0)	0 (0.0)	0 (0.0)	0 (0.0)	1 (50.0)	0 (0.0)	1 (100.0)	1 (100.0)	14 (4.5)
No pain	147 (94.8)	82 (90.1)	12 (92.3)	11 (57.9)	13 (92.9)	0 (0.0)	1 (100.0)	2 (100.0)	1 (50.0)	8 (100.0)	0 (0.0)	0 (0.0)	277 (89.9)
Unable to assess	1 (0.6)	4 (4.4)	0 (0.0)	1 (5.3)	0 (0.0)	1 (100.0)	0 (0.0)	0 (0.0)	0 (0.0)	0 (0.0)	0 (0.0)	0 (0.0)	7 (2.3)
**Day 2**	**N= 138**	**N= 87**	**N= 13**	**N= 19**	**N= 14**	**N= 1**	**N= 1**	**N= 2**	**N= 2**	**N= 8**	**N= 1**	**N=1**	**N= 287**
Patient reported	6 (4.3)	1 (1.2)	0 (0.0)	2 (10.5)	0 (0.0)	0 (0.0)	0 (0.0)	0 (0.0)	0 (0.0)	0 (0.0)	0 (0.0)	0 (0.0)	9 (3.1)
Parent reported	1 (0.7)	0 (0.0)	0 (0.0)	0 (0.0)	0 (0.0)	0 (0.0)	0 (0.0)	0 (0.0)	0 (0.0)	0 (0.0)	0 (0.0)	0 (0.0)	1 (0.3)
Clinician reported	4 (2.9)	0 (0.0)	0 (0.0)	3 (15.8)	0 (0.0)	0 (0.0)	0 (0.0)	0 (0.0)	0 (0.0)	0 (0.0)	0 (0.0)	1 (100.0)	8 (2.8)

No pain	117 (84.8)	80 (92.0)	9 (69.2)	12 (63.2)	11 (78.6)	0 (0.0)	0 (0.0)	1 (50.0)	0 (0.0)	7 (87.5)	0 (0.0)	0 (0.0)	237 (82.6)
Unable to assess	10 (7.2)	6 (6.9)	4 (30.8)	2 (10.5)	3 (21.4)	1 (100.0)	1 (100.0)	1 (50.0)	2 (100.0)	1 (12.5)	1 (100.0)	0 (0.0)	32 (11.1)
**Day 3**	**N=105**	**N=75**	**N=11**	**N=17**	**N=8**	**N=1**	**N=1**	**-**	**-**	**N=5**	**N=1**	**N=1**	**N=225**
Patient reported	3 (2.9)	0 (0.0)	0 (0.0)	1 (5.9)	0 (0.0)	0 (0.0)	0 (0.0)	-	-	0 (0.0)	0 (0.0)	0 (0.0)	4 (1.8)
Parent reported	0 (0.0)	2 (2.7)	0 (0.0)	0 (0.0)	0 (0.0)	0 (0.0)	0 (0.0)	-	-	0 (0.0)	0 (0.0)	0 (0.0)	2 (0.9)
Clinician reported	0 (0.0)	0 (0.0)	0 (0.0)	1 (5.9)	0 (0.0)	0 (0.0)	0 (0.0)	-	-	0 (0.0)	0 (0.0)	0 (0.0)	1 (0.4)
No pain	91 (86.7)	65 (86.7)	8 (72.7)	9 (52.9)	7 (87.5)	1 (100.0)	0 (0.0)	-	-	5 (100.0)	0 (0.0)	1 (100.0)	187 (83.1)
Unable to assess	11 (10.5)	8 (10.7)	3 (27.3)	6 (35.3)	1 (12.5)	0 (0.0)	1 (100.0)	-	-	0 (0.0)	1 (100.0)	0 (0.0)	31 (13.8)

Abbreviations: ECMO: extracorporeal membrane oxygenation

Supplementary Table 15: Skin Complications

	Vascular access devices	Gastrointestinal devices	Respiratory/ airway	Drains	Urinary	Epidural infusion	Regional/ local infusion	Cardiac pacing wires	Cardiac lines	Subcutaneous infusion	ECMO/ cardiac lines	Ventricular assist device	Total
	N (%)	N(%)	N (%)	N (%)	N (%)	N (%)	N (%)	N (%)	N (%)	N (%)	N (%)	N (%)	N (%)
**Day 0**	**N= 223**	**N= 112**	**N= 16**	**N= 25**	**N= 20**	**N= 1**	**N= 1**	**N= 2**	**N= 2**	**N= 8**	**N=1**	**N=1**	**N=412**
Skin tear	1 (0.4)	0 (0.0)	0 (0.0)	0 (0.0)	0 (0.0)	0 (0.0)	0 (0.0)	0 (0.0)	0 (0.0)	0 (0.0)	0 (0.0)	0 (0.0)	1 (0.2)
Irritant contact dermatitis	3 (1.3)	4 (3.6)	0 (0.0)	0 (0.0)	0 (0.0)	0 (0.0)	0 (0.0)	0 (0.0)	0 (0.0)	0 (0.0)	0 (0.0)	0 (0.0)	7 (1.7)
Pressure injury	2 (0.9): Stage 1	2 (1.8): Stage 1	0 (0.0)	0 (0.0)	0 (0.0)	0 (0.0)	0 (0.0)	0 (0.0)	0 (0.0)	0 (0.0)	0 (0.0)	0 (0.0)	2 (0.4)
**Day 1**	**N= 184**	**N=105**	**N= 16**	**N= 23**	**N= 20**	**N= 1**	**N= 1**	**N= 2**	**N= 2**	**N= 8**	**N= 1**	**N= 1**	**N=364**
Irritant contact dermatitis	2 (1.1)	0 (0.0)	0 (0.0)	0 (0.0)	1	0 (0.0)	0 (0.0)	0 (0.0)	0 (0.0)	0 (0.0)	0 (0.0)	0 (0.0)	3 (0.8)
Pressure injury	2 (0.9): Stage1	0 (0.0)	0 (0.0)	0 (0.0)	0 (0.0)	0 (0.0)	0 (0.0)	0 (0.0)	0 (0.0)	0 (0.0)	0 (0.0)	0 (0.0)	2 (<0.1)
**Day 2**	**N= 138**	**N= 87**	**N= 13**	**N= 19**	**N= 14**	**N= 1**	**N= 1**	**N= 2**	**N= 2**	**N= 8**	**N= 1**	**N= 1**	**N= 287**
Maceration	1 (0.7)	0 (0.0)	0 (0.0)	0 (0.0)	0 (0.0)	0 (0.0)	0 (0.0)	0 (0.0)	0 (0.0)	0 (0.0)	0 (0.0)	0 (0.0)	1 (0.3)
Irritant contact dermatitis	1 (0.7)	2 (2.3)	0 (0.0)	0 (0.0)	0 (0.0)	0 (0.0)	0 (0.0)	0 (0.0)	0 (0.0)	0 (0.0)	0 (0.0)	0 (0.0)	3 (1.0)
Skin tear	1 (0.7)	0 (0.0)	0 (0.0)	0 (0.0)	0 (0.0)	0 (0.0)	0 (0.0)	0 (0.0)	0 (0.0)	0 (0.0)	0 (0.0)	0 (0.0)	1 (0.3)
Skin stripping	1 (0.7)	0 (0.0)	0 (0.0)	0 (0.0)	0 (0.0)	0 (0.0)	0 (0.0)	0 (0.0)	0 (0.0)	0 (0.0)	0 (0.0)	0 (0.0)	1 (0.3)
Pressure injury	2 (1.4): Stage1	0 (0.0)	0 (0.0)	0 (0.0)	0 (0.0)	0 (0.0)	0 (0.0)	0 (0.0)	0 (0.0)	0 (0.0)	0 (0.0)	0 (0.0)	2 (0.7)
**Day 3**	**N= 105**	**N= 75**	**N= 11**	**N= 17**	**N= 8**	**N= 1**	**N=1**	**N=0**	**N=0**	**N=5**	**N=1**	**N=1**	**N=225**
Maceration	1 (1.0)	0 (0.0)	0 (0.0)	0 (0.0)	0 (0.0)	0 (0.0)	0 (0.0)	0 (0.0)	0 (0.0)	0 (0.0)	0 (0.0)	0 (0.0)	1 (0.4)
Irritant contact dermatitis	1 (1.0)	0 (0.0)	0 (0.0)	0 (0.0)	0 (0.0)	0 (0.0)	0 (0.0)	0 (0.0)	0 (0.0)	0 (0.0)	0 (0.0)	0 (0.0)	1 (0.4)
Skin stripping	2 (1.9)	0 (0.0)	0 (0.0)	0 (0.0)	0 (0.0)	0 (0.0)	0 (0.0)	0 (0.0)	0 (0.0)	0 (0.0)	0 (0.0)	0 (0.0)	2 (0.9)

Pressure injury	2 (1.9): Stage1 & Stage2	0 (0.0)	0 (0.0)	0 (0.0)	0 (0.0)	0 (0.0)	0 (0.0)	0 (0.0)	0 (0.0)	0 (0.0)	0 (0.0)	0 (0.0)	2 (0.9)

Abbreviations: ECMO: extracorporeal membrane oxygenation
